# Prognostic and clinicopathological value of Beclin-1 expression in hepatocellular carcinoma: a meta-analysis

**DOI:** 10.1186/s12957-018-1465-8

**Published:** 2018-08-14

**Authors:** Zhiqiang Qin, Xinjuan Yu, Mei Lin, Jinkun Wu, Shupei Ma, Ning Wang

**Affiliations:** 1 Department of Pathology, School of Basic Medicine, Medical College, Qingdao University, No. 308 Ningxia Road, Qingdao, 266071 Shandong People’s Republic of China; 2 Central Laboratories, Qingdao Municipal Hospital, Qingdao, 266071 Shandong People’s Republic of China; 3 Department of Hematology, Qingdao Municipal Hospital, Qingdao, 266011 Shandong People’s Republic of China

**Keywords:** Beclin-1, Hepatocellular carcinoma (HCC), Clinicopathological factors, Prognosis, Meta-analysis

## Abstract

**Background:**

The abnormal expression of Beclin-1 has recently been investigated in a variety of tumors. However, previous studies have obtained contradicting results regarding the clinical and prognostic value of Beclin-1 in hepatocellular carcinoma (HCC). We performed a meta-analysis to clarify the prognostic value of Beclin-1 and its correlations with clinical pathological parameters in HCC.

**Methods:**

Relevant studies were systematically retrieved from PubMed, EMBASE, China National Knowledge Infrastructure (CNKI), Wan Fang and Chinese VIP databases. We used the Newcastle-Ottawa scale (NOS) to estimate the quality of the involved studies.

**Results:**

Ten eligible studies with 1086 HCC patients were included in this study. Our results showed that decreased Beclin-1 expression in HCC related to histological grade [poor-undifferentiated vs. well-moderate: odds ratio (OR) = 2.34, 95% confidence interval (CI) = 1.65–3.32, *P* < 0.00001]. The pooled hazard ratio (HR) (HR = 1.43, 95% CI = 1.17–1.75, *P* = 0.0004) indicated that decreased Beclin-1 expression correlated with poor overall survival (OS).

**Conclusions:**

This meta-analysis indicated that decreased Beclin-1 expression might relate to poor differentiation and unfavorable outcome in HCC.

**Electronic supplementary material:**

The online version of this article (10.1186/s12957-018-1465-8) contains supplementary material, which is available to authorized users.

## Background

Hepatocellular carcinoma (HCC) is a principal cause of human cancer death worldwide [1]. Thus far, surgical excision is one of the most effective treatments for HCC [2]. Most early diagnosed HCC patients are treated by surgical excision. However, the post-operative recurrence remains high [3], and the 5-year overall survival (OS) is currently only approximately 18% [4]. Therefore, it is essential to identify an effective biomarker to predict the prognosis of HCC.

Autophagy is an intracellular catabolic process by which cytoplasmic proteins and organelles are delivered to lysosomes and subsequently degraded and recycled [5]. An increasing number of publications have shown that autophagy is closely related to the occurrence and progression of tumors [6]. However, the roles of autophagy in these processes are still controversial. Previous research reported that autophagy might mitigate metabolic stress and increase genomic stability to inhibit tumor development [7]. However, Ma et al. [8] showed that autophagy enhanced the resistance of glioblastoma to chemotherapy. At present, the impact of autophagy in HCC is also a subject of debate [9].

In 1998, Liang et al. [10] identified and cloned the mammalian homolog of yeast Atg6/Vps30 gene, namely, Beclin-1, which is located on human chromosome 17q21. It is a key regulator of autophagy. It induces autophagy by participating in autophagosome formation and endosome maturation, which are major steps of autophagy [11]. Abnormal expression of Beclin-1 was shown to relate to the occurrence and prognosis of breast cancer, gastric cancer, and lymphoma [12]. However, controversial results have been obtained in HCC. Yue et al. [13] found that Beclin-1+/− mice were more prone to develop malignant tumors, including HCC, than wild-type mice. Qiu et al. [14] showed that Beclin-1 expression was correlated with liver cirrhosis, Edmondson grades and vascular invasion. Ding et al. [15] showed that positive Beclin-1 expression was related to favorable outcome in HCC patients, while Wu et al. [16] demonstrated that Beclin-1 expression was not related to prognosis and any clinicopathological factors investigated in HCC. Hence, we conducted a meta-analysis to evaluate the clinical and prognostic value of Beclin-1 in HCC.

## Methods

### Literature search

A systematic literature search was carried out separately by two investigators (Zhiqiang Qin and Xinjuan Yu) in the English databases PubMed and EMBASE and the Chinese databases China National Knowledge Infrastructure (CNKI), Wan Fang and Chinese VIP, with an end date of 30 September 2017. Search terms were “Beclin-1” OR “beclin 1” OR “BECN1” OR “ATG6” AND “hepatocellular” OR “liver” OR “hepatic” AND “carcinoma” OR “tumor” OR “neoplasm” OR “cancer.” In addition, we manually retrieved the references of relevant reviews and the included literatures.

### Criteria for inclusion and exclusion

The following inclusion criteria were required for studies to be eligible: (1) were published in English or Chinese, and full-text articles can be retrieved; (2) were retrospective cohort studies; (3) had proven diagnosis of HCC in humans; (4) detected Beclin-1 protein expression by immunohistochemistry (IHC); (5) odds ratio (OR) or hazard ratio (HR) and 95% confidence interval (CI) on Beclin-1 expression and clinicopathological factors or OS could be obtained. Any study that met the following exclusion criteria was excluded: (1) non-original studies, such as review, case reports, letter to editors, or conference abstracts; (2) laboratory studies, such as studies on animal or cancer cell lines; (3) duplication of previous publications.

### Quality assessment

Newcastle-Ottawa scale (NOS), a recommended methodological quality assessment tool, was used to estimate the quality of the eligible literature [17]. Two investigators (Zhiqiang Qin and Xinjuan Yu) conducted the assessment independently. When disagreement occurred, the two investigators had a discussion or the third reviewer (Mei Lin) was recruited until consensus was reached. A study with a score ≥ 6 was graded as a high-quality study and others were graded as low quality [18].

### Data extraction

Two investigators (Zhiqiang Qin and Xinjuan Yu) independently extracted data from the included studies. Disagreements regarding data extraction were crosschecked until achieving consensus. The following information were extracted: surname of the first author, publication year, country, number of patients, gender, age range, antibody dilution, evaluation methods, cut-off value, percentage of decreased Beclin-1 expression, histopathological parameters, and HR and 95% CI of Beclin-1 expression for OS. When HR and its 95% CI were not explicitly given in the article, we performed the calculation using data provided by the literature according to the methods reported by Tierney et al. [19].

### Statistical analysis

The current study was conducted based on the Preferred Reporting Items for Systematic Reviews and Meta-Analyses (PRISMA) guidelines [20] (Additional file 1). Pooled ORs and 95% CIs were used to evaluate the associations between Beclin-1 expression and clinicopathological factors. Pooled HR and 95% CI were applied to estimate the effect of Beclin-1 expression on OS. Interstudy heterogeneity was assessed by chi-squared test (*Q* test) and *I*^2^ test (range from 0-100%). A *P* value (Q test) > 0.10 and *I*^2^ ≤ 50% indicated no significant heterogeneity. In this case, we used the fixed effect model. When heterogeneity was evident (*P* value ≤ 0.10 or *I*^2^ > 50%), we used the random effect model. Subgroup analysis and sensitivity analysis were performed to explore sources of heterogeneity. Publication bias was evaluated by funnel plots. All analyses were carried out using Review Manager (version: 5.3, Cochrane Informatics and Knowledge Management Department, http://tech.cochrane.org/revman/download). *P* < 0.05 was considered statistically significant.

## Results

### Selection of included articles

**Fig. 1:**
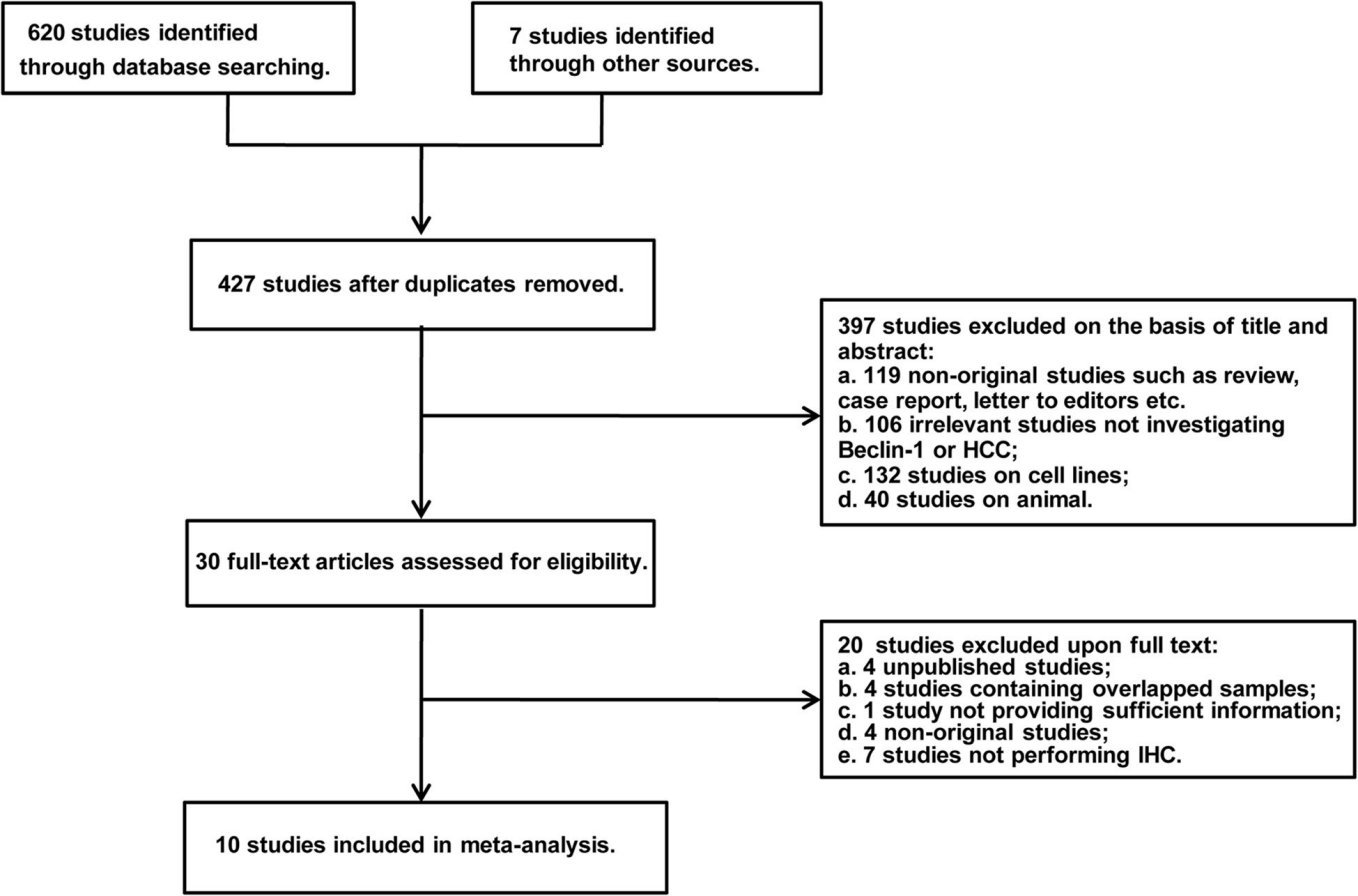
Flow chart of study selection

Six hundred and twenty potential articles were initially retrieved from relevant electronic databases, and 7 articles were obtained from a manual search of references. A total of 200 repeated documents were excluded. After reviewing the titles and abstracts, 119 non-original articles, 106 irrelevant articles, and 172 laboratory studies on animals or cell lines were removed. The remaining 30 articles were further investigated by attentively reading the full text. Twenty articles were then excluded due to not fulfilling the inclusion criteria or fulfilling the exclusion criteria. Ultimately, 10 articles with a total of 1086 HCC patients were eligible for further analysis. The selection steps and the reasons for exclusion are summarized in Fig. 1.

### Characteristics and quality assessment of studies

The basic characteristics of the 10 included articles are listed in Table 1. In all, the involved studies consisted of 8 English studies [14–16, 21–25] and 2 Chinese studies [26, 27]. These studies were published between 2008 and 2016, and the number of participants ranged from 35 to 300. Of the 10 studies, 7 were from China, 2 from Egypt, and 1 from Korea. All the patients included had proven pathological diagnosis of HCC. IHC staining was used to investigate Beclin-1 expression, and immunoreactive score (IRS) was used to assess Beclin-1 expression. The percentage of decreased Beclin-1 expression ranged from 18.52 to 94.21%. Five studies provided information on OS. However, HR and 95% CI could only be obtained in four studies.

**Table 1:** Characteristics of the included studies

References	Year	Country	No. of patients	Gender (M/F, *n*)	Age range (years)	Method	Antibody dilution	Counting method	Cut-off staining	Reduced Beclin-1 expression (%)	OS data provided
Ding [15]	2008	China	300	252/48	NR	IHC	1:50	IRS*	10%	205/300 (68.33)	Yes
Kang [21]	2013	China	50	47/3	28–71	IHC	NR	IRS	4	11/50 (22.00)	No
Lee [22]	2013	Korea	190	158/32	29–76	IHC	NR	IRS	6	179/190 (94.21)	Yes
Guo [26]	2013	China	54	39/15	33–75	IHC	1:150	IRS	3	10/54 (18.52)	No
Qiu [14]	2014	China	103	85/18	21–79	IHC	1:100	IRS	8	81/103 (78.64)	Yes
Wu [16]	2014	China	156	143/13	NR	IHC	1:100	IRS	6	83/156 (53.21)	Yes
Osman [23]	2015	Egypt	65	51/14	40–74	IHC	1:100	IRS*	10%	32/65 (49.23)	No
Yang [27]	2015	China	50	39/11	26–74	IHC	NR	IRS	3	11/50 (22.00)	No
Al-Shenawy [24]	2016	Egypt	35	20/15	23–75	IHC	1:350	IRS	1	18/35 (51.43)	No
Zhou [25]	2016	China	83	69/14	NR	IHC	1:100	NR	NR	45/83 (54.22)	Yes

*M* male, F female, *NR* not reported, *IHC* immunohistochemistry, *IRS* immunoreactive score, IHC expression was evaluated integrating proportion and intensity of positive staining, IRS* IHC expression was evaluated by percent positivity, *OS* overall survival

The quality of the 10 eligible studies was evaluated by NOS. The scores were all ≥ 6 points (Table 2). This indicated that all the included studies were high-quality studies.

**Table 2:** Newcastle-Ottawa scale for quality assessment

Study	Selection				Comparability	Outcome			Total
	Exposed cohort	Non-exposed cohort	Ascertainment of exposure	Outcome of interest	Control for factor	Assessment of outcome	Follow-up long enough	Adequacy of follow-up	score
Ding [15]	*	*	*	*	**	*	*	*	9
Kang [21]	*	*	*	*	*	*	*	*	8
Lee [22]	*	*	*	*	**	*	*		8
Guo [26]	*	*	*	*	**	*	*	*	9
Qiu [14]	*	*	*	*	**	*	*	*	9
Wu [16]	*	*	*	*	**	*	*	*	9
Osman [23]	*	*	*	*	*	*	*	*	8
Yang [27]	*	*	*	*	**	*	*	*	9
Al-Shenawy [24]	*	*	*	*	**	*	*	*	9
Zhou [25]	*	*		*	*	*	*		6

*A study can be awarded a maximum of one star for each numbered item within the selection and outcome categories. A maximum of two stars can be given for comparability. http://www.ohri.ca/programs/clinical_epidemiology/oxford.asp

### Beclin-1 expression and clinicopathological parameters

**Fig. 2:**
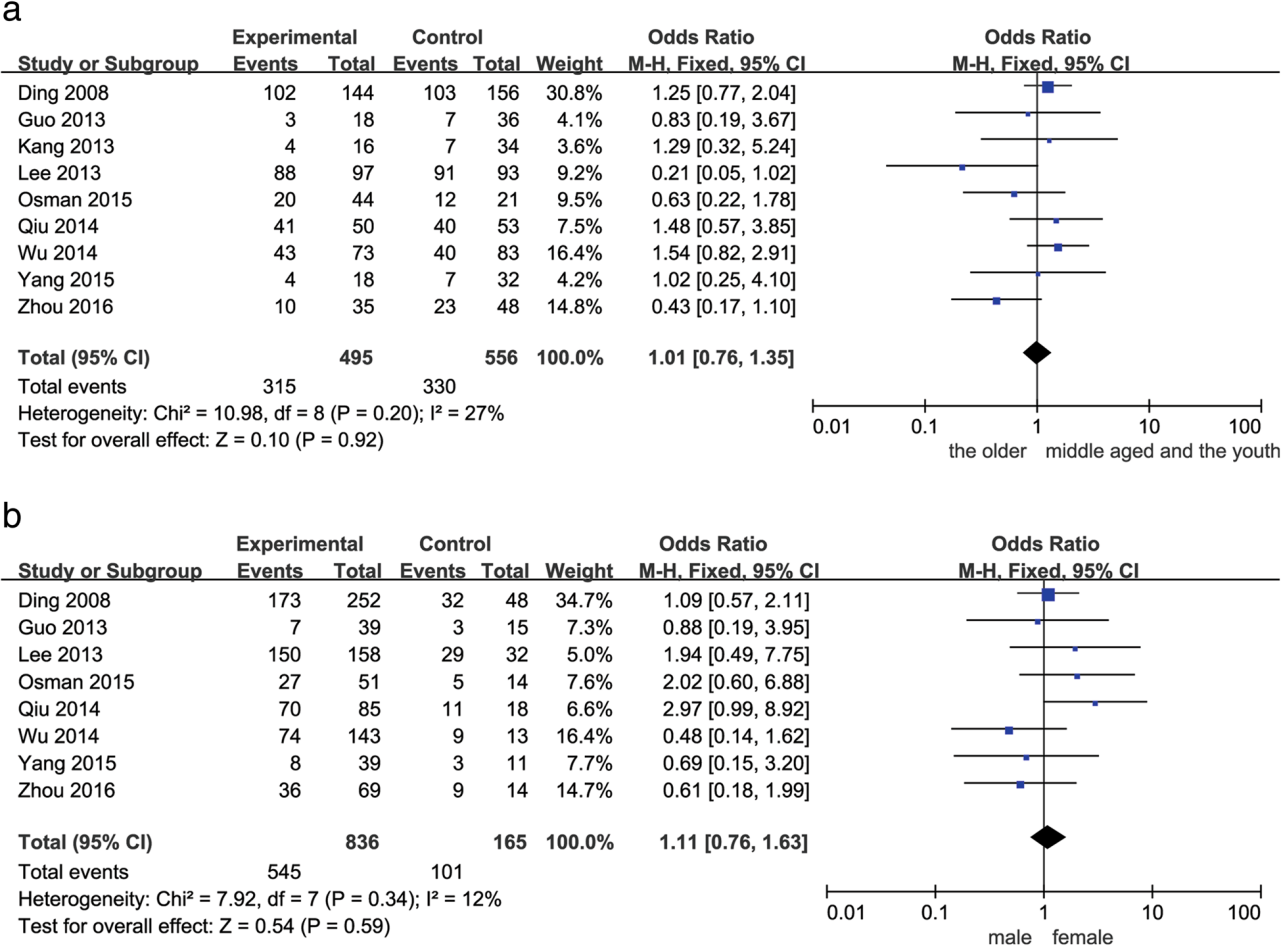
Forest plot of studies assessing the relationship between Beclin-1 expression and **a** age and **b** gender

**Fig. 3:**
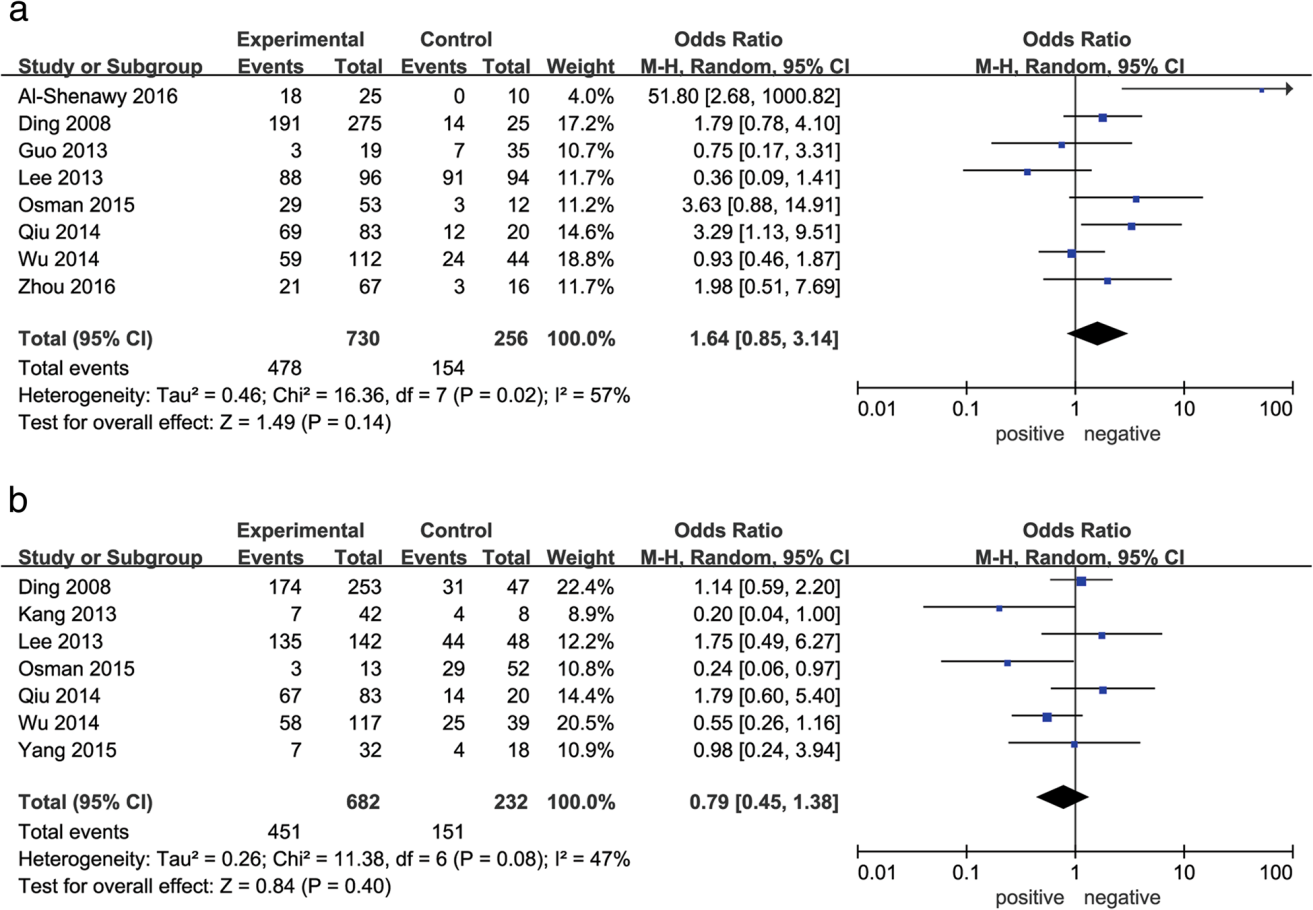
Forest plot of studies assessing the relationship between Beclin-1 expression and **a** liver cirrhosis and **b** HBsAg

**Fig. 4:**
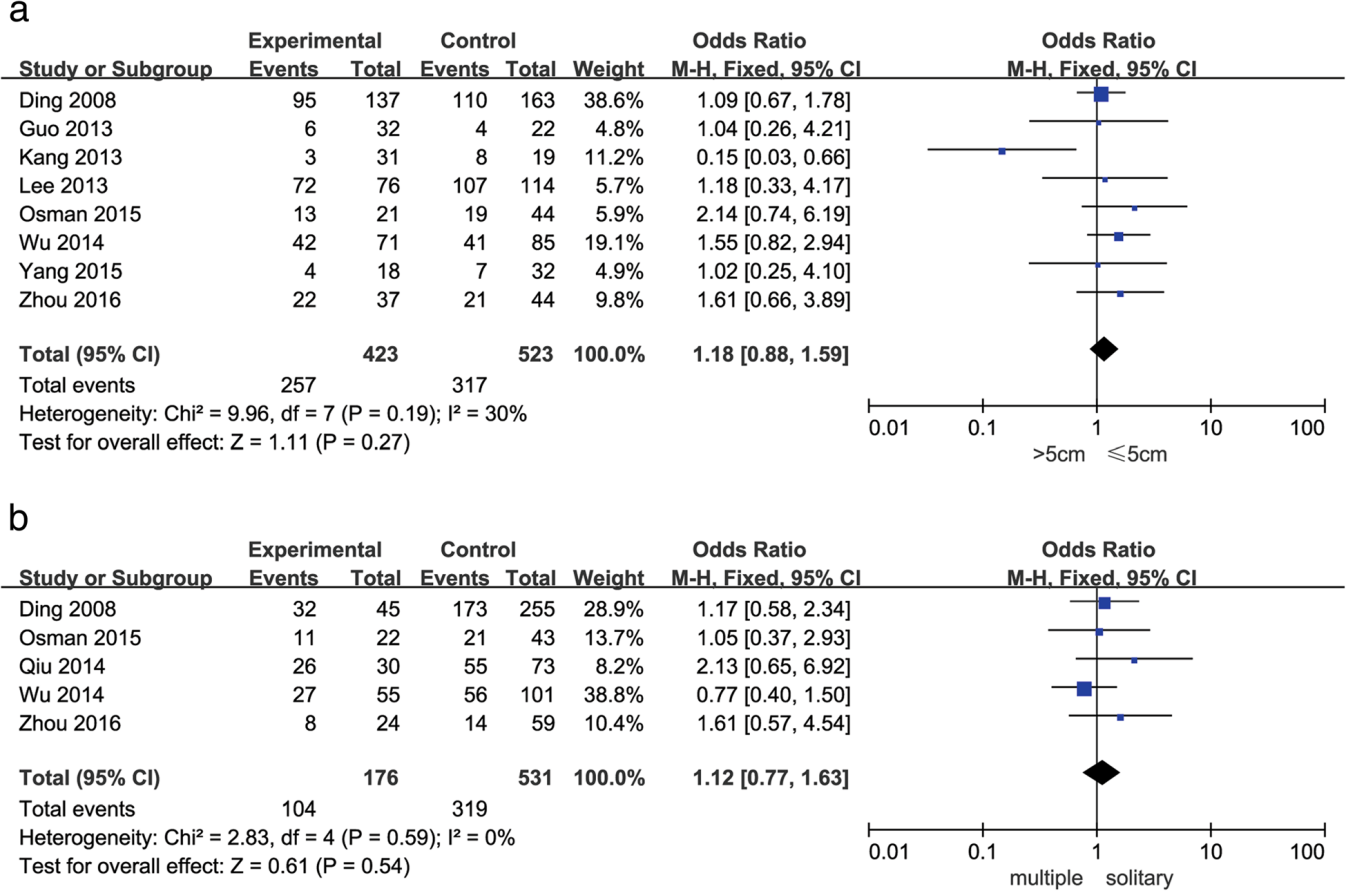
Forest plot of studies assessing the relationship between Beclin-1 expression and **a** tumor size and **b** tumor number

**Fig. 5:**
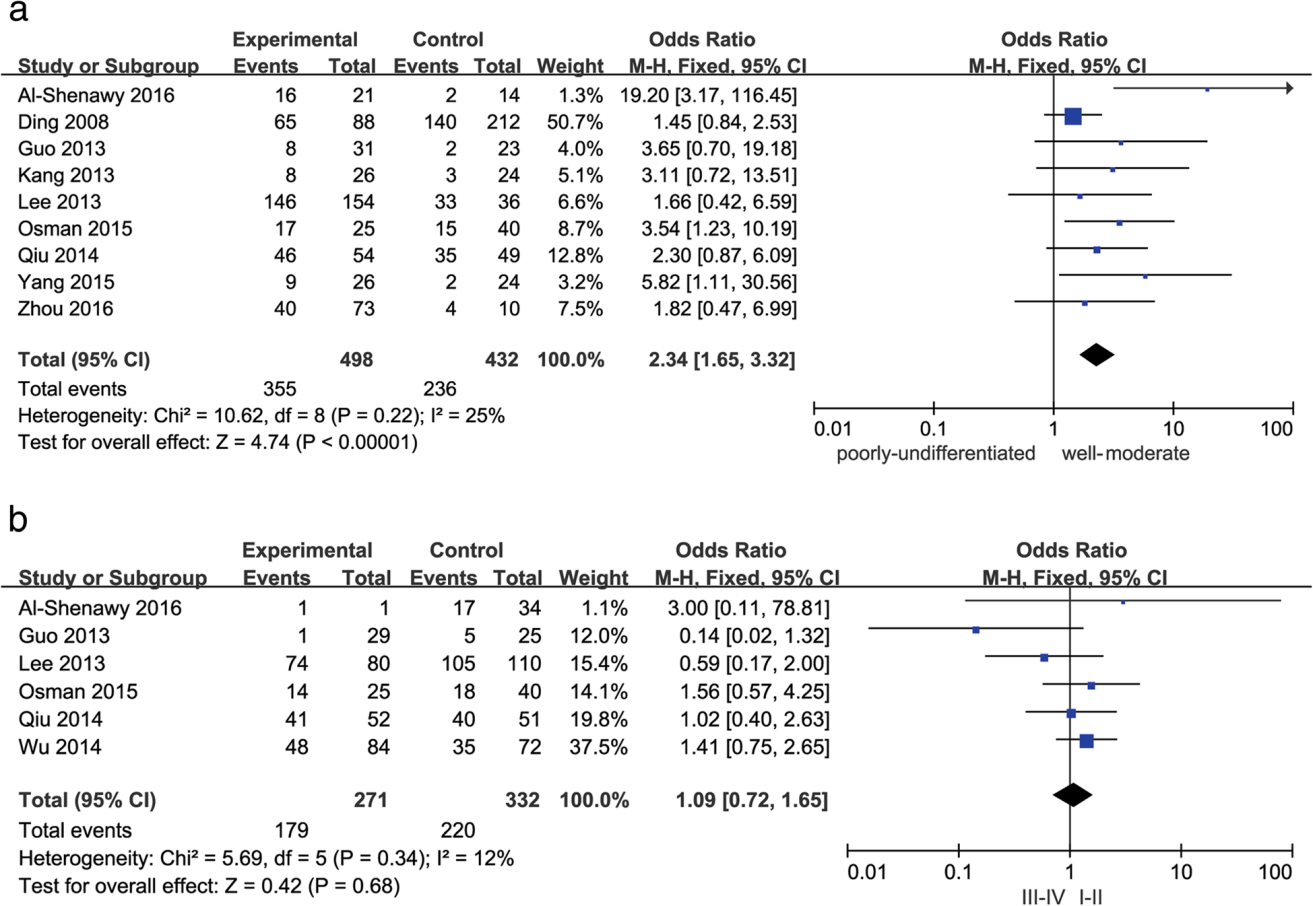
Forest plot of studies assessing the relationship between Beclin-1 expression and **a** histological grade and **b** TNM stage

As shown in Figs. 2, 3, 4 and 5, decreased Beclin-1 expression was significantly correlated with histological grade (poorly undifferentiated vs. well-moderated: OR = 2.34, 95% CI = 1.65–3.32, *P* < 0.00001), with slight heterogeneity (*P* = 0.22, *I*^2^ = 25%). However, decreased Beclin-1 expression was not related to age (older vs. middle aged and youth: OR = 1.01, 95% CI = 0.76–1.35, *P* = 0.92), gender (male vs. female: OR = 1.11, 95% CI = 0.76–1.63, *P* = 0.59), liver cirrhosis (positive vs. negative: OR = 1.64, 95% CI = 0.85–3.14, *P* = 0.14), HBsAg (positive vs. negative: OR = 0.79, 95% CI = 0.45–1.38, *P* = 0.40), tumor size (> 5 cm vs. ≤ 5 cm: OR = 1.18, 95% CI = 0.88–1.59, *P* = 0.27), tumor number (multiple vs. solitary: OR = 1.12, 95% CI = 0.77–1.63, *P* = 0.54), or TNM stage (III–IV vs. I–II: OR = 1.09, 95% CI = 0.72–1.65, *P* = 0.68). Considerable interstudy heterogeneity was observed in the analyses of the correlation between Beclin-1 expression and liver cirrhosis (*P* = 0.02, *I*^2^ = 57%) or HBsAg (*P* = 0.08, *I*^2^ = 47%). The analyses of Beclin-1 expression and other clinicopathological factors exhibited slight or no heterogeneity.

### Beclin-1 expression and OS

**Fig. 6:**
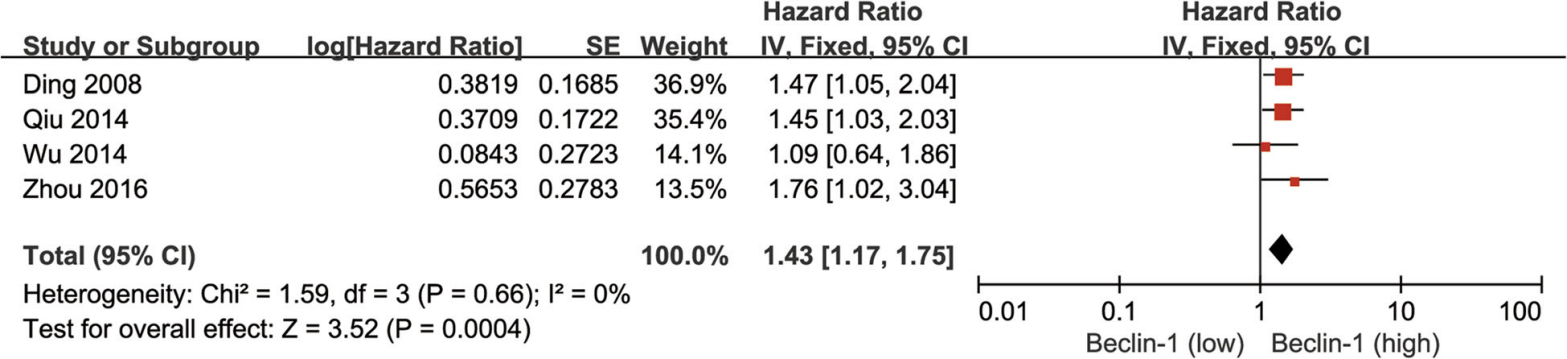
Forest plot of studies assessing the relationship between Beclin-1 expression and OS in HCC patients

Four reports that included 642 HCC patients were eligible to investigate the correlation between reduced Beclin-1 expression and OS. As shown in Fig. 6, decreased Beclin-1 expression in HCC was related to poor OS (Beclin-1 low vs. Beclin-1 high: HR = 1.43, 95% CI = 1.17–1.75, *P* = 0.0004). There was no heterogeneity in this analysis (*P* = 0.66, *I*^2^ = 0%).

### Sensitivity analysis

We conduct a sensitivity analysis by excluding one study in turn. In the study of the correlation between Beclin-1 expression and liver cirrhosis, Beclin-1 expression was lower in patients with cirrhosis than in those without cirrhosis (OR = 1.95, 95% CI = 1.04–3.66, *P* = 0.04) after removing Lee et al.’s study [22], and heterogeneity was still obvious (*P* = 0.06, *I*^2^ = 50%). Except Lee et al.’s study, none of the individual studies evidently impacted the outcomes of the current study.

### Subgroup analysis

Remarkable heterogeneity was observed in the meta-analysis of the correlation between Beclin-1 expression and liver cirrhosis or HBsAg. To explore the sources of the heterogeneities, we performed subgroup analysis based on geographic region (Table 3). Beclin-1 was irrelevant to liver cirrhosis of HCC patients from both Asia (OR = 1.27, 95% CI = 0.72–2.25, *P* = 0.42) and Egypt (OR = 10.03, 95% CI = 0.74–136.23, *P* = 0.08). Evident heterogeneity existed in Egyptian HCC patients (*P* = 0.10, *I*^2^ = 63%), but not in Asian HCC patients (*P* = 0.12, *I*^2^ = 42%). Beclin-1 was not related to the status of HBsAg in Asian HCC patients (OR = 0.91, 95% CI = 0.61–1.34, *P* = 0.62) with no evident heterogeneity (*P* = 0.15, *I*^2^ = 38%). However, Beclin-1 expression was significantly decreased in Egyptian HBsAg negative HCC patients (OR = 0.24, 95% CI = 0.06–0.97, *P* = 0.04).

**Table 3:** Subgroup analysis of liver cirrhosis and HBsAg

Subgroups	No. of studies	Pooled OR (95% CI)	*P* value	Heterogeneity	
				*I* ^2^	*P* value
Liver cirrhosis					
Asia	6	1.27 (0.72–2.25)	0.42	42%	0.12
Egypt	2	10.03 (0.74–136.23)	0.08	63%	0.10
HBsAg					
Asia	6	0.91 (0.61–1.34)	0.62	38%	0.15
Egypt	1	0.24 (0.06–0.97)	0.04	–	–

*HBsAg* hepatitis B surface antigen, *OR* odds ratio, *CI* confidence interval

### Publication bias

**Fig. 7:**
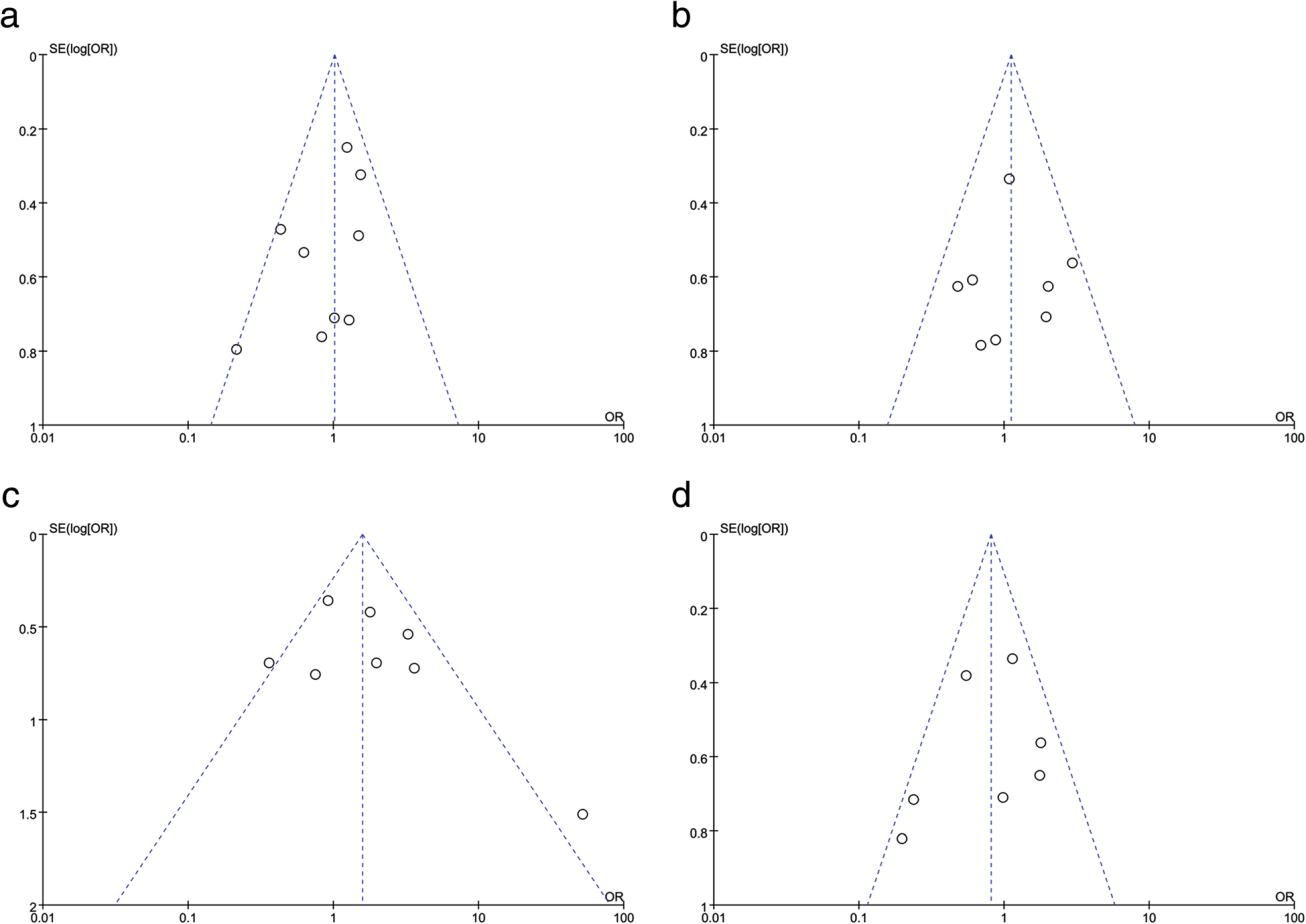
Funnel plot for assessing publication bias of the relationship between Beclin-1 expression and **a** age, **b** gender, **c** liver cirrhosis, and **d** HBsAg

**Fig. 8:**
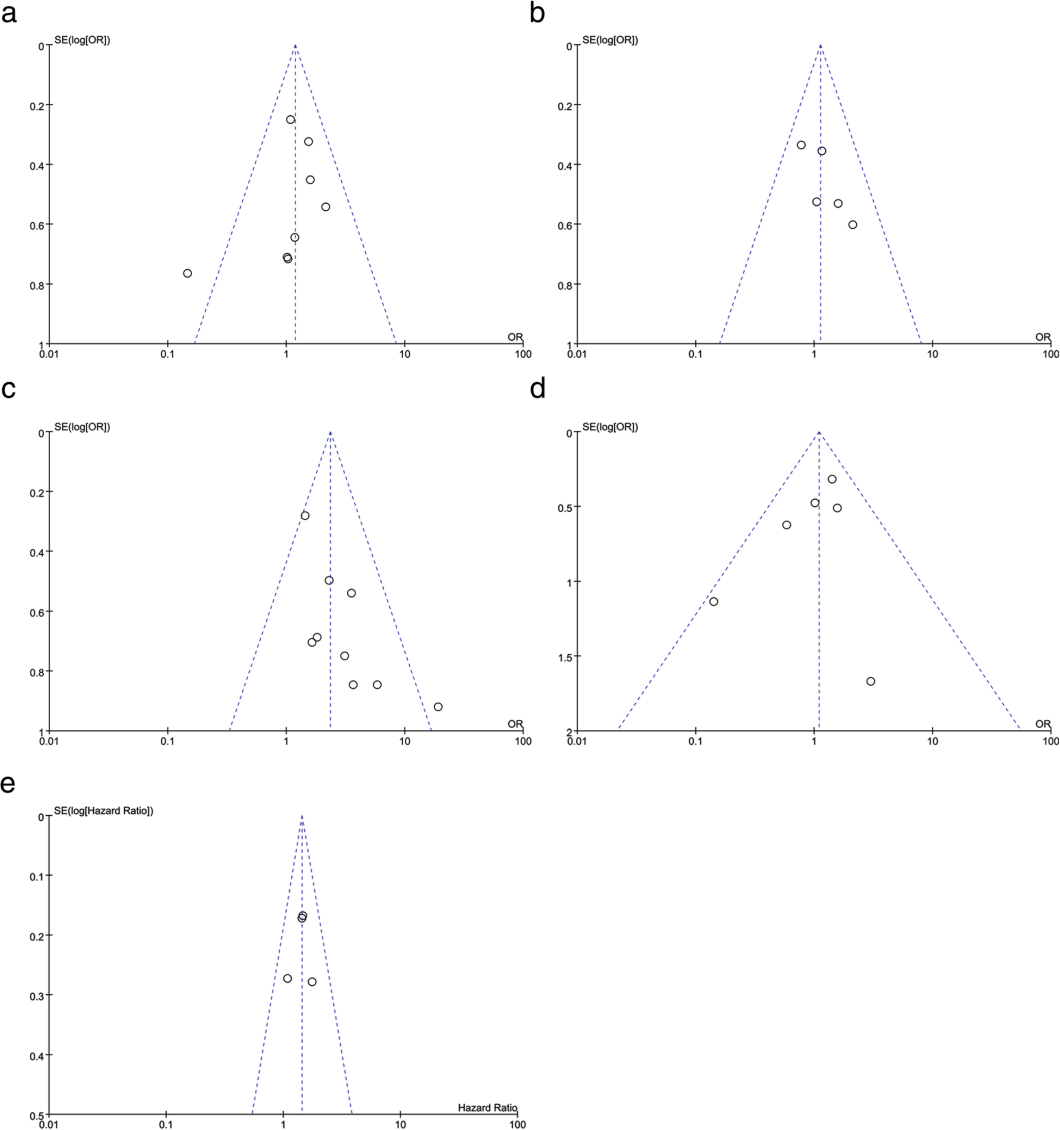
Funnel plot for assessing publication bias of the relationship between Beclin-1 expression. **a** Tumor size. **b** Tumor number. **c** Histological grade. **d** TNM stage. **e** OS

Funnel plots of all included studies were symmetrical (Figs. 7 and 8), indicating that there was no significant publication bias present in the current study.

## Discussion

In this meta-analysis, the electronic databases PubMed, EMBASE, CNKI, Wan Fang, and Chinese VIP were systematically searched, and 10 studies with 1086 HCC patients matched the inclusion criterion. The current study demonstrated that decreased Beclin-1 expression related to poor differentiation in HCC. This was in accordance with Xia et al.’s meta-analysis [28], which showed that reduced Beclin-1 expression was related to poor differentiation in gastric cancer. Consistent with the results obtained in cholangiocarcinoma, bladder, and ovarian cancers [29–31], we demonstrated that decreased Beclin-1 expression was associated with unfavorable outcome in HCC patients. These suggested that decreased Beclin-1 might have tumor suppressor function in HCC and indicated that decreased Beclin-1 expression may signify the poor prognosis of HCC. Hence, activation of autophagy may improve the prognosis of HCC patients. Previous studies demonstrated that Beclin-1 downregulated angiogenesis and proliferation of malignant cells [32] and postponed cell cycle progression [33]. In addition, Mathew et al. [34] demonstrated that Beclin-1 could limit genome instability. These may contribute to the tumor suppressor function of Beclin-1. In contrast, other groups discovered that increased Beclin-1 expression was correlated with unfavorable outcome in oral squamous cell carcinoma [35], nasopharyngeal carcinoma [36], and pancreatic ductal adenocarcinoma [37]. This discrepancy might be because the role of autophagy on tumor progression was tissue specific.

Significant heterogeneity existed in the analysis of the correlation of Beclin-1 expression and liver cirrhosis or HBsAg. Thus, we performed sensitivity analysis and subgroup analysis to discover the sources of the heterogeneities. In sensitivity analysis, Beclin-1 expression was significantly decreased in HCC patients with liver cirrhosis compared to those without liver cirrhosis, but heterogeneity was still obvious after excluding Lee et al.’s study [22]. The rate of decreased Beclin-1 expression was 94.21% in Lee et al.’s study [22], which was higher than the other 7 studies (18.52–78.64%). We hypothesized that the high rate of decreased Beclin-1 expression in Lee et al.’s study might alter the result of analysis. Subgroup analysis based on geographic region showed that Beclin-1 expression was irrelevant to liver cirrhosis of HCC patients from both Asia and Egypt. This suggested that regional difference did not influence the association between Beclin-1 expression and liver cirrhosis in this study. The heterogeneity mainly existed in Egyptian HCC patients. This may be because only two studies from Egypt were involved in this study.

Until now, this study has been the first comprehensive and systematic meta-analysis investigating the clinical and prognostic value of autophagic-related protein Beclin-1 in HCC. This study solved the debate regarding whether Beclin-1 was correlated with clinicopathological factors and prognosis of HCC patients. However, several limitations in this study should be acknowledged. First, although Beclin-1 expression in all of the included reports was detected by IHC, the evaluation methods and cut-off values were diverse. This may be a potential source of heterogeneity. Second, in addition to Beclin-1, other autophagy-related proteins, such as LC3 or Atg-9, can also be investigated to clarify the role of autophagy in HCC. Third, of the 10 included studies, 8 were from Asia and 2 were from Egypt. Studies from other areas were not available. This may cause publication bias and make it difficult to indicate the correlation of Beclin-1 and clinicopathological factors or prognosis among HCC patients from Europe and America. Fourth, studies with positive results are more likely to be published than those reporting negative results. This may also cause publication bias and might potentially contribute to the limited geographical regions involved in the current study. Finally, only four studies were eligible for analyzing the prognostic role of Beclin-1 in the current study. Therefore, more studies are needed to verify the results obtained by the current study.

## Conclusion

In conclusion, this study demonstrated that decreased Beclin-1 expression might relate to poor differentiation and unfavorable outcome in HCC. With regard to the shortcomings of this meta-analysis, we expect studies with larger sample sizes to verify our results.

## Additional file

Additional file 1: PRISMA 2009 Checklist. (DOC 64 kb)

## Abbreviations

CI: Confidence interval
CNKI: Chinese databases China National Knowledge Infrastructure
HCC: Hepatocellular carcinoma
HR: Hazard ratio
IHC: Immunohistochemistry
IRS: Immunoreactive score
NOS: Newcastle-Ottawa scale
OR: Odds ratio
OS: Overall survival
PRISMA: Preferred Reporting Items for Systematic Reviews and Meta-Analyses
